# Evaluation of least significant changes of pulse contour analysis-derived parameters

**DOI:** 10.1186/s13613-019-0590-z

**Published:** 2019-10-11

**Authors:** Hugues de Courson, Loic Ferrer, Grégoire Cane, Eric Verchère, Musa Sesay, Karine Nouette-Gaulain, Matthieu Biais

**Affiliations:** 1 Department of Anesthesiology and Critical Care, Pellegrin Bordeaux University Hospital, 33000 Bordeaux, France; 2 Biostatistics Unit, Institut Curie, U900, Hôpital René Huguenin Saint-Cloud, Saint-Cloud, France; 3 INSERM, U12-11, Laboratoire de Maladies Rares: Génétique et Métabolisme (MRGM), Univ. Bordeaux, Bordeaux, France; 4 Adaptation cardiovasculaire à l’ischémie, U1034, Univ. Bordeaux, 33600 Pessac, France; 5 Department of Anaesthesiology and Critical Care Pellegrin, Hôpital Pellegrin, CHU de Bordeaux, 33076 Bordeaux Cedex, France

**Keywords:** Pulse contour, Stroke volume, Pulse pressure variation, Stroke volume variation, Precision

## Abstract

**Background:**

Many maneuvers assessing fluid responsiveness (minifluid challenge, lung recruitment maneuver, end-expiratory occlusion test, passive leg raising) are considered as positive when small variations in cardiac index, stroke volume index, stroke volume variation or pulse pressure variation occur. Pulse contour analysis allows continuous and real-time cardiac index, stroke volume, stroke volume variation and pulse pressure variation estimations. To use these maneuvers with pulse contour analysis, the knowledge of the minimal change that needs to be measured by a device to recognize a real change (least significant change) has to be studied. The aim of this study was to evaluate the least significant change of cardiac index, stroke volume index, stroke volume variation and pulse pressure variation obtained using pulse contour analysis (ProAQT^®^, Pulsion Medical System, Germany).

**Methods:**

In this observational study, we included 50 mechanically ventilated patients undergoing neurosurgery in the operating room. Cardiac index, stroke volume index, pulse pressure variation and stroke volume variation obtained using ProAQT^®^ (Pulsion Medical System, Germany) were recorded every 12 s during 15-min steady-state periods. Least significant changes were calculated every minute.

**Results:**

Least significant changes statistically differed over time for cardiac index, stroke volume index, pulse pressure variation and stroke volume variation (*p* < 0.001). Least significant changes ranged from 1.3 to 0.7% for cardiac index, from 1.3 to 0.8% for stroke volume index, from 10 to 4.9% for pulse pressure variation and from 10.8 to 4.3% for stroke volume variation.

**Conclusion:**

To conclude, the present study suggests that pulse contour analysis is able to detect rapid and small changes in cardiac index and stroke volume index, but the interpretation of rapid and small changes of pulse pressure variation and stroke volume variation must be done with caution.

## Introduction

Cardiac output monitoring is recommended in many situations, both in the intensive care unit and the operating room [1–3]. Pulse contour analysis is one of the most frequently used technologies [4]. Several devices propose an external cardiac output calibration (using transpulmonary thermodilution or echocardiography, for example) and other devices use internal calibration using complex algorithms. Such devices provide cardiac index (CI) and stroke volume index (SVI) estimations and are used in this way to evaluate variations induced by therapeutic or diagnostic interventions. An important limitation of pulse contour analysis technology is its high sensitivity to changes in systemic vascular resistance making external calibration frequently necessary in patients with low systemic vascular resistance or after changes in vasopressor dosage [5–7]. The main advantage of this technology is the ability to assess cardiac output continuously and in real time.

Many maneuvers assessing fluid responsiveness need precise and rapid assessment of cardiac output and stroke volume. For example, passive leg raising induces an increase in venous return followed by an increase in stroke volume in preload responsive patients. An increase in cardiac output ≥ 10 ± 2% discriminated patients who will respond or not to fluid administration [8]. When using end expiratory occlusion test, the best cut-off value is a cardiac output increase of 5% [9, 10]. A stroke volume increase ≥ 6% after a minifluid challenge of 100 mL predicts fluid responsiveness [11]. Finally, it has been recently proposed that a decrease ≥ 30% of stroke volume after a recruitment maneuver was able to discriminate fluid responder [12]. Even though the accuracy of pulse contour-derived cardiac output has been extensively studied, to our knowledge, the least significant change (LSC) (i.e., the minimal change that needs to be measured by a device to recognize a real change) has not yet been studied. Furthermore, pulse contour analysis provides continuous assessment of pulse pressure variation (PPV) and stroke volume variation (SVV). Because of increasing limitations of the use dynamic indices, some authors proposed to evaluate changes in PPV and SVV following maneuvers such as transient increase in tidal volume or minifluid challenges to predict fluid responsiveness [13]. Moreover, variations of these indices discriminating responders and non-responders are very low (2 to 3.5%) and no data are available on the capacity of pulse contour analysis to detect such small variations. Although several studies have demonstrated the reliability of such tests using pulse contour, several questions remain unanswered regarding the ability of this technology to track such small CI, SVI, PPV or SVV variations. Some authors have pointed out the small number of patients included in these studies and the possibility of recruitment biases [14]. Measurement of the LSC appears necessary to determine the clinical applicability of this type of tests at the bedside [15–18].

The primary aim of the present study was to evaluate the LSC of CI and SVI derived from pulse contour analysis. Secondary aims were to evaluate the least significant changes of PPV and SVV derived from pulse contour analysis (ProAQT^®^, Pulsion Medical System, Germany).

## Materials and methods

### Patients

This study obtained the approval of the Institutional Review Board (Comité d’Ethique pour la Recherche en Anesthésie-Réanimation, IRB-00010254-2018-054). The patients were informed about the inclusion of their anonymized health data in our database (Written informed consent was waived by the Institutional Review Board).

Inclusion criteria were as follows: patients older than 18 years scheduled for neurosurgery, equipped with radial arterial catheter and cardiac output monitor, surgery in the supine position and the absence of arrhythmia.

### Perioperative management

Patients were monitored with non-invasive blood pressure, SpO_2_ and ECG. Total intravenous anesthesia was used by target-controlled infusion of remifentanil and propofol. Patients were mechanically ventilated using a volume-control mode with a tidal volume of 6–8 ml/kg of ideal body weight, respiratory rate was adjusted to maintain normocapnia, positive expiratory pressure was set between 3 and 6 cmH_2_O, F_I_O_2_ was adjusted to maintain pulse oximetry above 95% and inspiratory/expiratory ratio was 0.5

### Hemodynamic monitoring

A radial arterial catheter inserted after the induction of anesthesia was connected to a specific transducer (ProAQT^®^, Pulsion Medical System) for SVI, SVV and PPV monitoring. The initial value of cardiac output was estimated with a proprietary algorithm performing an “auto-calibration”. Stroke volume was then determined by pulse contour analysis. PPV and SVV were continuously displayed on the Pulsioflex^®^ monitor. SVI, CI, PPV and SVV measurements using ProAQT^®^ are an average during the last 12 s and are updated every second. SVI, CI, PPV and SVV are displayed beat-to-beat as a “sliding average” of 12 s. When extracting data via a USB port to an Excel file, values were displayed every 12 s.

### Study design

After the “auto-calibration”, data were continuously recorded every 12 s on the Pulsioflex^®^ monitor, and extracted using USB port as an Microsoft^®^ Excel file for statistical analysis. Recording started at least 20 min after the onset of induction of anesthesia. Data were recorded during hemodynamic stability (defined as a change in mean arterial pressure and heart rate less than 5%). In practice, we made these recordings during the placement of surgical drapes. Patients with hemodynamic instability requiring a decrease (or an increase) in anesthesia drug dosage, fluid infusion or administration of vasopressors, were excluded. Another exclusion criterion was any change in ventilatory setting by the physician in charge of the patient.

### Statistical analysis

Data are expressed as mean (95% confidence interval) or median (5–95th percentiles) according to variable distribution. LSC is the minimum change that needs to be measured by a device to recognize a real change. LCS was calculated for cardiac index, stroke volume index, PPV and SVV as previously described [15–17]:

$${\text{Coefficient}}\;{\text{of}}\;{\text{variation}}\;\left( {\text{CV}} \right) = {\text{standard}}\;{\text{deviation}}/{\text{mean}}\;{\text{of}}\;{\text{measurements}}$$

$${\text{Coefficient}}\;{\text{error}}\left( {\text{CE}} \right) = {\text{CV}}/\surd n \, \left( {n = {\text{number}}\;{\text{of}}\;{\text{measurements}}\;{\text{per}}\;{\text{patient}}} \right)$$

Least significant changes (LSC) = The LSC can be described by the following equation:

$${\text{LSC}} = {\text{CE}} \times 1. 9 6\times \surd 2.$$

To analyze the LSC of pulse contour ,data were recorded every 12 s, so five measurements were analyzed every minute. LSC calculation included 2 measurements for 30 s, 3 measurements for 45 s, 5 measurements for 1 min, 10 measurements for 2 min, 15 measurements for 3 min, etc. Average LSC on 15 min was calculated as the mean of individual LSC. The same applies for LSC calculation at each time-point. For example, LSC at 30 s was calculated as the mean of individuals LSC calculated at 30 s. To compare the LSC averages at each minute, and to take into account repeated measurements, we performed an ANOVA for repeated measurements. If one of the means differed statistically from the others, we performed a Tukey test with Bonferroni correction to take into account the multiplicity of tests.

Because LSC was based on standard deviation calculation, distribution of CI, SVI, PPV or SVV had to follow a normal distribution. According to the central limit theorem, as the number (size) of the independent variable increases, the more likely to obtain a normal distribution of the sample. A minimal number of 30 is currently accepted to assume a normal distribution. Thus, the number of subjects should be ≥ 30. After taking into account uninterpretable data or exclusion, we considered that 50 subjects were needed for this study.

Statistical analysis was performed using Medcalc (software 16.4.3; Mariakerke, Belgium) and R Development Core Team ([2008]. R: A language and environment for statistical computing; R Foundation for Statistical Computing, Vienna, Austria. ISBN 3-900051-07-0, URL).

## Results

### Patients’ characteristics

Fifty patients were included. Their main characteristics are shown in Table 1. No patient was excluded because of hemodynamic instability. Hemodynamic variables at baseline are shown in Table 2.

**Table 1:** Population’s characteristics

Characteristics	
Age (years)	59 ± 13
Gender	
Male/female	20 (40)/30 (60)
Body mass index (kg/m^2^)	26.5 ± 5.3
ASA classification	
ASA 1	9 (18)
ASA 2	10 (20)
ASA 3	30 (60)
ASA 4	1 (2)
Surgery	
Cerebral tumor	37 (74)
Other	13 (26)
Surgery duration (h)	2.8 ± 1.1
Tidal volume (ml)	423 ± 55
Tidal volume (ml/kg/ideal weight)	7 ± 1
PEEP (cmH_2_O)	5 ± 2
Respiratory rate (cycles/min)	14 ± 2
FiO2 (%)	41 ± 9

Data are expressed as mean ± standard deviation or as number (percentage)

*ASA* American Society of Anesthesiologists, *PEEP* positive end expiratory pressure, *F*_*I*_*O*_*2*_ fraction inspired oxygen

**Table 2:** Hemodynamic baseline values

Variables	
Heart rate (bpm)	63 [58–79]
Mean arterial pressure (mmHg)	77 [71–90]
Cardiac index (l/min/m^2^)	2.7 [2.4–3.1]
Stroke volume index (ml/m^2^)	40 [38–46]
Pulse pressure variation (%)	7 [6–11]
Stroke volume variation (%)	10 [7–14]

Values are expressed as median [interquartile range 25–75%]

### Evaluation of least significant changes

**Fig. 1:**
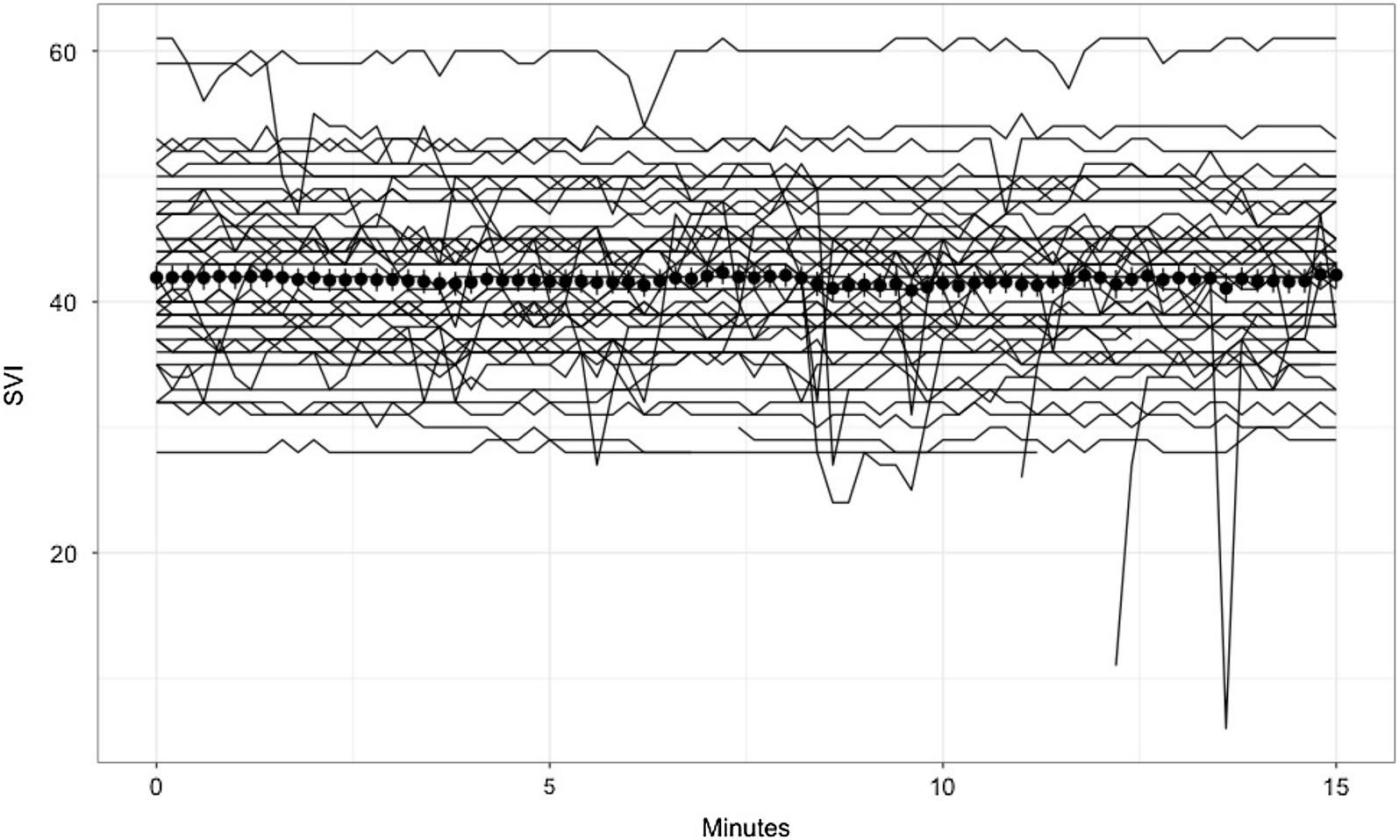
Spaghetti plots: Individual values (solid lines), mean (circle) and standard deviation of stroke volume index (SVI) during the 15 min of recording

**Fig. 2:**
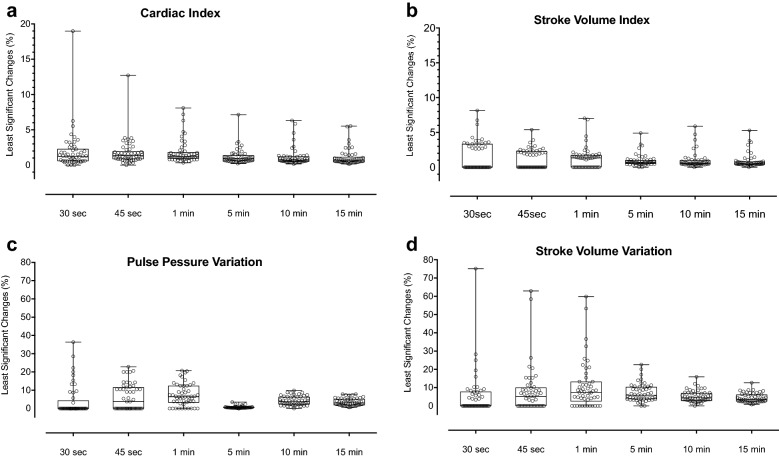
**a** Representation of the least significant changes of cardiac index at predefined times: 30 s (s), 45 s, 60 s, 5 min (min), 10 min and 15 min). Individual values (circles) and box plot (lines corresponding to median, upper and lower bars represent 5–95th percentiles). **b** Representation of the least significant changes of stroke volume index at predefined times: 30 s (s), 45 s, 60 s, 5 min (min), 10 min and 15 min). Individual values (circles) and box plot (lines corresponding to median, upper and lower bars represent 5–95th percentiles). **c** Representation of the least significant changes of pulse pressure variation at predefined times: 30 s (s), 45 s, 60 s, 5 min (min), 10 min and 15 min). Individual values (circles) and box plot (lines corresponding to median, upper and lower bars represent 5–95th percentiles). **d** Representation of the least significant changes of stroke volume variation at predefined times: 30 s (s), 45 s, 60 s, 5 min (min), 10 min and 15 min). Individual values (circles) and box plot (lines corresponding to median, upper and lower bars represent 5–95th percentiles)

Individual values, mean and standard error of SVI of all patients over time are shown in Fig. 1. Over 15 min, mean LSC was 1.1% for cardiac index, 1% for stroke volume index, 6.5% for PPV and 6.5% for SVV. The LSC values during the 15-min recording period are shown in Fig. 2a–d. For SVI, CI, PPV and SVV the LSC statistically differed between minutes (*p* < 0.001). Among the 50 patients, 12 had at least one SVI value that was more than 10% different from the initial value. During the 15 min of recording, 3.8% of the SVI values showed a variation of more than 10% from the initial value.

### LSC and prediction/identification of fluid responsiveness

Table 3 shows the LSC of CI, SVI, PPV and SVV for different tests to predict fluid responsiveness. LSC of CI and SVI for the identification of fluid responsiveness are shown in Table 3.

**Table 3:** Prediction and identification of fluid responsiveness: least significant change (LSC), timing and proposed cut off value of changes in cardiac index (CI), stroke volume index (SVI), pulse pressure variation (PPV) and stroke volume variation (SVV)

Tests to predict fluid responsiveness				
Tests	Variable	Timing (s)	Cut off	LSC
Occlusion test	CI	15/30	5%	1.2% (0.2–5.0)
MiniFluid	SVI	60	6%	1.3% (0–4.2)
	PPV	60	2% (absolute change)	8.9% (0–26%)
	SVV	60	2% (absolute change)	7.5% (0–35%)
Passive leg raising	CI	90	10–15%	1.3% (0.5–5.6)
Tidal volume challenge	PPV	60	3.5% (absolute change)	8.9% (0–26%)
	SVV	60	2.5% (absolute change)	7.5% (0–35%)

Identification of fluid responsiveness			
Variable	Timing (min)	Cut off (%)	LSC
SVI	10	10–15	0.5% (0.2–3.5)
SVI	15	10–15	0.5% (0.2–3.4)
CI	10	10–15	0.7% (0.4–4.1)
CI	15	10–15	0.7% (0.3–3.3%)

Results for LSC are median (5–95th percentiles)

## Discussion

This study demonstrates that when using pulse contour analysis such as ProAQT^®^: (i) the LSC of CI and SVI is low making the detection of small and rapid changes in CI and SVI possible and (ii) LSC of both PPV and SVV is higher making the detection of small and rapid changes in PPV and SVV more hazardous.

### LSC signification

The LSC is defined as the minimal change that needs to be measured by a device to recognize a real change. In the hemodynamic area, LSC of cardiac index and stroke volume index has been studied for transpulmonary thermodilution and transthoracic echocardiography [15–18]. To our knowledge, no data were available for cardiac index, stroke volume index, PPV and SVV obtained using pulse contour analysis. Many studies evaluated the accuracy of stroke volume index (absolute value and trending) and the accuracy of PPV and SVV obtained with pulse contour technology. Pulse contour analysis does not provide measurements of SVI, but its estimation. Thus, the absolute value of SVI may be inaccurate, particularly in vasoplegic patients [5–7, 19–21]. This potential low accuracy does not preclude a good precision [22, 23].

### Identification of fluid responsiveness

Classical definition of fluid responsiveness is a CI or SVI increase of at least 10–15% after volume expansion administered over 10–15 min. We found a LSC of CI and SVI very much below the 10–15% threshold, making it possible to identify the effect of fluid administration. Several studies suggest that the magnitude of changes in SVV or PPV induced by volume expansion could identify fluid responsiveness [13, 24]. A decrease of 1.4%–2.5% of the absolute value of PPV or SVV after volume expansion has been proposed to discriminate responders. These thresholds are higher than LSC (therefore usable) when PPV and SVV values are less than 25%, which is usually the case.

### Prediction of fluid responsiveness

As the ability of SVV and PPV to predict the responsiveness during protective ventilation is limited, new approaches of fluid responsiveness have been proposed. Changes in CI and SVI have been studied following postural maneuvers (passive leg raising, Trendelenburg position), end-expiratory occlusion test, minifluid challenge and following an increase in intrathoracic pressure (lung recruitment maneuvers) [8, 9, 11, 12, 25, 26]. The best threshold values for changes in SVI, analyzed a few seconds to 2 min after the maneuver, were between 5 and 30%. These values exceeded the 2% LSC calculated for SVI using pulse contour, so these tests can be used with pulse contour analysis such as ProAQT^®.^

Several studies demonstrated that small changes in PPV or SVV (2% to 3.5% of the absolute value) following minifluid challenge and transient increase in tidal volume are able to predict fluid responsiveness [13, 27]. According to our results, these thresholds are higher than LSC (therefore usable) when initial PPV and SVV values are less than 18–20%. This should be taken into account when carrying out and analyzing these tests.

### Study limitations

This study has some limitations. First, study was performed using ProAQT^®^’s algorithm and our results cannot be extrapolated to other algorithms. Second, we may wonder why patients can be considered hemodynamically stable over 15 min. Indeed, some subjects may have had a variation in MAP of more than 5%, which may lead to a discussion of the notion of hemodynamic stability. However, from a population point of view, the evolution of MAP was still within the 5% range. In addition, this situation reflects the real life during which physicians are led to use preload dependence tests and the purpose of our work was to evaluate the LSC for clinical application at the bedside. Moreover, even if some subjects may have experienced greater variations than others during this period described as stable, this would be responsible for a decrease in precision and therefore an increase in the LSC, which remains sufficiently low to validate the use of the device under study as part of the evaluation of the fluid responsiveness. Third, the calculation of the LSC under the study conditions is debatable because we analyzed serial measurements of multiple values but not repeated measurements of a single value [23, 28]. Furthermore, LSC values may appear artificially reduced using numerous repeated measurements. However, we found that very few SVI measurements exhibited a variation of more than 10% from the initial value. Finally, the present study included patients without arrhythmia, not receiving vasopressors or inotropes and positioned in the supine position. Extrapolation to other study populations should be very cautious.

## Conclusion

The present study suggests that during 15 min of stable recording, sudden but brief changes in SVI values can occur. However, the average LSC value is compatible with the detection of rapid and brief variations of SVI. The interpretation of rapid and small changes of PPV and SVV must be interpreted with caution.

## Data Availability

Data are available from the authors on reasonable request.

## Abbreviations

CE: coefficient error
CI: cardiac index
CV: coefficient of variation
LSC: least significant changes
PPV: pulse pressure variation
SVV: stroke volume variation
SVI: stroke volume index
